
JOURNAL INFORMATION
==============================
NLM Title Abbreviation: Northwest Med Surg J
NLM Journal ID: 0020727

The North-Western Medical and Surgical Journal

Publisher: Biomedical Journal Digitization Project

ARTICLE INFORMATION
==============================
PMCID: PMC9973939
PMID: 37320812
Article version: 1
Subjects: Miscellaneous Items

Miscellaneous Items

Print publication date: 1857 Oct
Volume: 6
Volume Series: New Ser
Issue: 10
First page: 483
Last page: 483
License: This work is free of known copyright restrictions. For more information, please see PMC Back Issue Digitization.
License URL: https://creativecommons.org/publicdomain/mark/1.0/

==============================

Science for 1821,') which certainly deserves the full attention of all medical practitioners, and which, if confirmed by experience, may have the most beneficial results."

### MISCELLANEOUS ITEMS.

*Handsome Bequest.*

A valuable addition has been made to the Geological Cabinet of Yale College, of all the geological drawings of the late Dr. Mantell, of England, the distinguished author of the Wonders of Geology. Dr. Mantell died in 1852, bequeathing these drawings to the College. They were forwarded from Europe by Dr. Mantell's son, and have arrived at New Haven.

*Singular Case of Triplets.*

Dr. A. S. McGregor, of Gasconade Ferry, Mo., in a letter to the editor of this journal, says, " On the 10th of August, 1856, Mrs. G——, of this County, was delivered of a still-born child. Twenty-one days after this, she gave birth to a second, and in the same length of time thereafter, to a third. The last two lived about six hours each. The mother is doing well, and is again pregnant.—*St. Louis Med. and Surg. Journal.*

Prof. Wittstein, a German Naturalist, has announced the discovery of Lactic Acid, heretofore considered of exclusive animal origin, in vegetables, especially in the peduncles of *Solanum dulcamara*, and in the liquid which dropped from freshly cut vine branches. It would seem the farther researches are carried, the fewer distinctions remain between vegetable and animal substances.—*Peninsular Jour. of Medicine.*

### TO THE MEDICAL PROFESSION.

The subscribers would call the attention of physicians to the annexed list of Fluid Extracts, which we have been induced to prepare, from the difficulty of obtaining such preparations of a reliable character, and to obviate the great inconvenience of being dependent on distant manufacturers for articles of every day use by physicians.
